# Respiratory function in healthy long-term meditators: a systematic review

**DOI:** 10.1186/s13643-023-02412-0

**Published:** 2024-01-02

**Authors:** L. J. U. Karunarathne, W. A. D. L. Amarasiri, A. D. A. Fernando

**Affiliations:** 1 Department of Physical Medicine, National Hospital of Colombo, Colombo, 00700 Sri Lanka; 2 Department of Physiology, Faculty of Medicine, University of Colombo, Colombo, 00800 Sri Lanka

**Keywords:** Long-term meditation, Healthy long-term meditators, Respiratory function, Respiratory rate, Spirometry, Meditation experience

## Abstract

**Background:**

There is a paucity of data on effects of long-term meditation on respiration. This systematic review summarized selected respiratory function parameters in healthy long-term meditators (LTMs) at rest, during meditation and their associations with meditation practice variables.

**Methods:**

A systematic search of PubMed, EMBASE (Ovid), Scopus, Proquest Dissertation and Thesis Global databases, CENTRAL, and Google Scholar was performed from year 1950 to August 15th, 2023. Keywords “meditation,” “long-term meditation,” and respiratory/pulmonary/lung function and spirometry were used. Controlled-trials and observational studies exploring respiratory parameters in healthy LTMs published in English were included. Two independent reviewers selected studies, extracted data, and assessed the quality of the evidence. The Joanna-Briggs Institute Critical Appraisal Tools and the Single-Case Reporting Guideline In BEhavioural Interventions Statement were used to assess the methodological quality of the included studies. This review followed the Preferred Reporting Items for Systematic Reviews and Meta-Analyses (PRISMA) guidelines. Effect estimates of some outcomes were synthesized using alternative methods and data for other outcomes synthesized narratively as a meta-analysis was not possible.

**Results:**

Nine studies comprising 3 case–control, 3 cross-sectional, and 3 single-subject study designs, involving 433 participants that met the eligibility criteria, were included. Two studies reported slower resting RR among LTMs compared to controls [SMD = − 2.98, 95% CI (− 4.48 to − 1.47), overall-effect (*z*-score) = 3.88 (*p* < 0.001), *I*^2^ = 69%] with similar trend reported in the third study (MD = − 1.6, *p* = 0.053). Three studies reported slower RR in LTMs during meditation compared to baseline. Slower resting RR and mean RR change during meditation compared to baseline significantly negatively associated with meditation experience. PEFR was significantly higher in LTMs than controls [MD = 1.67, 95% CI (0.19–3.15), *z*-score = 2.21 (*p* = 0.03)]. No significant difference was observed in tidal volume [SDM = 0.93, 95% CI (− 1.13 to 2.99), *z*-score = 0.89 (*p* = 0.37), *I*^2^ = 96%] and vital capacity [SDM = 1.25, 95% CI (− 0.45 to 2.95), *z*-score = 1.44 (*p* = 0.15), *I*^2^ = 94%] of LTMs compared to controls.

**Conclusions:**

Long-term meditation appears to be associated with slower baseline RR, and immediate reduction in RR during meditation, where greater practice amplifies the effects. Evidence on spirometry parameters in LTMs with ≥ 3 years of practice was limited.

**Supplementary Information:**

The online version contains supplementary material available at 10.1186/s13643-023-02412-0.

## Background

Meditation refers to a set of mental practices leading to an altered state of consciousness characterized by heightened alertness, expanded awareness, greater presence, and a more integrated sense of self [1]. Meditation, though practiced in search of spiritual achievements, has also been found to lead to significant beneficial health outcomes in healthy individuals, as well as in those with diseases [2, 3].

Meditation is thought to affect physiological responses involving various biological systems [1, 4]. Numerous studies have reported significant physiological changes including a reduction in metabolic activity [5, 6], resting heart rate [1], blood pressure [1, 7, 8], and sympathetic activity, and increase in parasympathetic activity [4, 9].

Attention to respiration is a central component of most meditative practices. Rhythmic breathing is a foundational component in breath-based meditation techniques, which guides practitioners towards a deep meditative, relaxed mental state. Therefore, respiration is considered a crucial factor for reaching the meditative state of consciousness, or “Samadhi” [10]. Respiration influences hemodynamic and autonomic parameters. The rate of respiration (RR) has been found to directly affect cardiovascular hemodynamics [11, 12] while a correlation between respiration and phasic parasympathetic vagal activity has been observed in some studies [13, 14]. Therefore, given its prominent role in formal meditation and centrality in body physiological processes, the study of respiratory function in relation to meditation would offer potential insight into the pathways by which contemplative/meditative practice may lead to this wide array of beneficial physiological changes.

Many studies exploring the short-term effects of various meditation techniques on respiratory parameters in healthy individuals have reported significant reductions in RR [8, 15], and minute volume (MV) and increase in tidal volume (TV) [15], peak expiratory flow rate (PEFR), and chest expansion [2]. If short-term meditation practice shows changes in respiratory function, one could anticipate that long-term meditation practice would show substantial changes as well. However, since there is a dearth of longitudinal studies exploring long-term meditation, it remains to be established whether distinctive respiratory function changes occur in LTMs because of their long-term practice experience. None of the previous systematic reviews in the literature has evaluated respiratory function changes in healthy LTMs with ≥ 3 years of meditation experience, where one study in search of evidence on meditative movements for cystic fibrosis patients reported that there is very limited evidence on the influence of meditative movements on respiratory function in healthy individuals [16]. Another recent meta-analysis on the efficacy of yogic interventions on pulmonary function and respiratory muscle strength parameters also highlighted the limited number of evidence available and the wide heterogeneity among the studies included [17]. In this context, this systematic review aimed to provide a comprehensive systematic evaluation of selected respiratory function parameters in healthy long-term meditators (LTMs) practicing any meditation technique which comes under the umbrella term “meditation.” The primary objective of this review was to assess selected respiratory function parameters (RR, lung volumes, lung capacities, and spirometry parameters) at rest in healthy adult LTMs with ≥ 3 years of meditation experience, compared to meditation-naïve (non-meditating) participants. The focus will be on elucidating differences in respiratory function among these groups. This review also summarizes selected respiratory function changes during meditation compared to baseline rest in healthy adult LTMs, to identify immediate responses during the practice of meditation in healthy LTMs and to investigate the influence of various meditation practice variables (e.g., cumulative meditation practice experience, total hours of sitting meditation) on selected respiratory function parameters in healthy LTMs.

The complexity of primary interest lies in understanding how selected respiratory function parameters in healthy LTMs differ from those in meditation-naïve individuals and whether meditation experience variables are associated with changes in these selected respiratory parameters irrespective of the type/technique of meditation they practice. Further, this review will contribute to the understanding of the dynamic interplay between meditation and respiratory function in healthy individuals providing evidence for future research and clinical use. Discovery of whether such meditation practices are capable of positively influencing respiratory function would be useful to develop meditation-based clinical interventions.

## Methods

This systematic review followed the standard recommended methodology and adhered to the Preferred Reporting Items for Systematic Reviews and Meta-Analyses (PRISMA) 2020 statement: an updated guideline for reporting systematic reviews [18]. The checklist of PRISMA reporting guidelines for this review can be found in Additional file 1 (Supplementary Table S1). Though a protocol was developed for this study (Additional file 2), the protocol was not prospectively registered.

### Search strategy

A systematic search of PubMed, EMBASE (Ovid), Scopus electronic databases, and Cochrane Central Register of Controlled Trials (CENTRAL) registry was performed from the year 1950 to 15th August 2023. In addition to the above, a gray literature search was performed in Google Scholar and Proquest Dissertation and Thesis Global database to access relevant gray literature. The search terms included “meditation,” “long-term meditation,” “long-term meditators,” “healthy,” AND keywords related to respiratory function (“respiratory function” OR “pulmonary function” OR “lung function” OR spirometry). All searches were limited to the English language. The complete search strategy for each database is presented in Additional file 3. We also performed a manual search of references included in the selected articles.

### Eligibility criteria

For inclusion in this review, studies had to be conducted with healthy LTMs and the reported mean years of meditation experience of the meditator group in the study had to be ≥ 3 years. A meditator with a mean practice experience of at least 3 years or more in a particular type of meditation, regardless of the daily routine practice and retreat experience, was considered a “long-term meditator” only for the purpose of selecting studies to be included in this review. The key criteria adopted for the inclusion and exclusion of studies according to the participants, interventions, comparisons, outcomes, and study design (PICOS) characteristics are presented in Table 1.

**Table 1:** Study selection criteria

Study component	Inclusion criteria	Exclusion criteria
**Participants**	Healthy, adult long-term meditators (LTMs) practicing any type of meditation technique denoted by the umbrella term “meditation.” (A long-term meditator was defined as a meditator with a mean practice experience of at least 3 years or more in a particular type of meditation, regardless of the daily routine practice and retreat experience for the purpose of selecting studies to be included in this review)	Studies involving unhealthy/diseased individuals, monks as “LTMs”, and those younger than 16 years and/or older than 70 years were excluded
**Study design**	Controlled trials and observational studies (cross-sectional, longitudinal, case–control, and cohort)	Articles not available in full-text form, reviews, case reports, news items, conference proceedings, and unpublished data were excluded
**Intervention and comparison**	Studies involving LTMs in any type of meditation technique, comparing selected respiratory function parameters at rest in healthy LTMs with matched controls, short-term meditators (STMs) or both, and/or studies comparing selected respiratory function parameters of LTMs during meditation with the same respiratory function parameters at rest, and studies which assessed the associations between selected respiratory function parameters and meditation practice variables were considered as eligible to be included in this review	Studies involving non-meditative relaxation techniques
**Outcome**	Studies investigated on selected respiratory function measures of healthy LTMs at rest and during meditation including rate of respiration (RR), spirometry parameters (forced expiratory volume in the first second (FEV_1_), ratio of forced expiratory volume in the first second/forced vital capacity (FEV_1_/FVC), peak expiratory flow rate (PEFR), tidal volume (TV), vital capacity (VC), and minute ventilation	Reports in which the practice experience of LTMs was unclear were not included

*LTMs* Long-term meditators, *STMs* Short-term meditators, *RR* Respiratory rate; *FEV*_*1*_ Forced expiratory volume in 1 s, *FEV*_*1*_*/FVC* Ratio of forced expiratory volume in the first second/forced vital capacity

### Study selection and data extraction

All identified records by search were imported into the Endnote library to remove duplications. All references which had the same title and author, and published in the same year or/and published in the same journal, were deleted. After duplication removal, all remaining references were screened for eligibility.

Data extraction was done using a specially designed data extraction form and the data were extracted by two independent reviewers (UK and LA). Essential information for screening, including authors’ names, publication year, journal, DOI/URL link, and the abstract was collected in the data extraction form.

Two independent reviewers (UK and LA) conducted the title/abstract screening and excluded those that did not report the outcome of interest. Two reviewers (UK and LA) independently went through the full-text articles, selected studies, extracted the data, and assessed the methodological quality of the selected articles. Two reviewers (UK and LA) went through the references listed in the selected articles to yield any reports that may have been missed in the initial search. All possible relevant records followed the same title/abstract and full-text screening against the eligibility criteria. Any disagreements were resolved by discussion with the third reviewer (DF) with agreement reached in all cases. The extracted data from the selected articles included authors, study setting, study design, year of publication, participant characteristics, comparison characteristics, sample size, respiratory outcome measures, and detailed method of outcome measures and findings related to respiratory function. In particular, data on selected respiratory function parameters (respiratory rate, minute ventilation, tidal volume, vital capacity, and spirometry parameters), the mean values, standard deviations, and sample sizes were extracted from the included studies. For further analysis, data were organized into an Excel spreadsheet.

### Data management and synthesis

Collected data were synthesized with the use of Review Manager (RevMan) software version 5.4.1. with random-effect analysis. All included studies underwent a comprehensive thorough evaluation. The analyses were complemented by computing effect estimates for selected respiratory function parameters. Data were summarized for each outcome variable with standardized mean difference (SMD) and mean difference (MD) where appropriate presenting 95% confidence interval (CI) ranges, overall effect size, and its significance level for each. Heterogeneity was categorized as low (*I*^2^ = ≤ 25%), moderate (*I*^2^ = 26–74%), and considerably high (*I*^2^ = ≥ 75%). Evidence on associations between selected respiratory function parameters and meditation practice variables was synthesized narratively.

### Quality assessment

The risk of bias and the methodological quality of the included studies were assessed independently by two authors (UK and LA). Any discrepancies were resolved by discussion with the third author (DF). For the quality assessment of the cross-sectional and case–control studies, the Joanna Briggs Institute (JBI) Critical Appraisal Tools [19] were utilized. Single-subject designed studies were reviewed according to the Single-Case Reporting Guideline In BEhavioural Interventions (SCRIBE) Statement [20].

In all quality assessment tools, each criterion was evaluated as “Yes”, “No”, or “Other” (unclear/ not applicable). An overall rating was provided for each study based on the items rated with an affirmative answer (“Yes” = 1, “No” = 0, “Other” = 0), and calculated the percentage of the total score. Accordingly, the quality score was determined by the range 67–100 (good), 34–66 (fair), and 0–33 (bad).

## Results

**Fig. 1:**
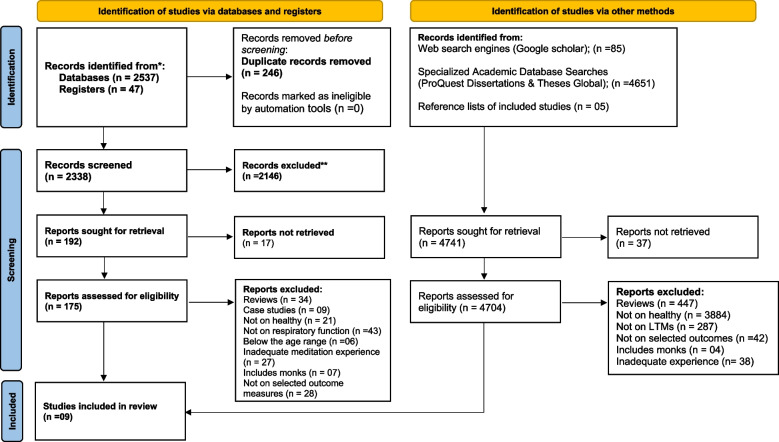
PRISMA flow chart of the study selection process

The initial search yielded an early pool of 7325 articles published in the English language from the selected databases, registers, and sources of gray literature. In total, 246 duplicate studies were removed from databases and registry search results, and 2338 records were screened for eligibility by 2 independent reviewers (UK and LA). Following the title and abstract screening, 2146 records from the database and registry search results and 4331 from the gray literature search were excluded. Out of the 192 records sought for retrieval from database and registry search, 175 full-text articles were checked for eligibility and 7 articles meeting the inclusion criteria were retained and included in the review. Additionally, 2 articles meeting the inclusion criteria were selected from the gray literature search. Finally, a total of 9 studies were included in the review. A complete flowchart of the study selection process is presented in Fig. 1.

### Study characteristics

Out of the 9 included studies, 3 were case–control (CC) studies, 3 were cross-sectional (CS) studies, and 3 adopted single-subject (SS) study design. None adopted longitudinal or randomized controlled designs. The sample sizes ranged from 7 to 105, while 6 out of the 9 studies had 7–40 participants [6, 21–25], 2 studies involved 60–100 participants [26, 27], and only 1 study had over 100 participants [28]. Characteristics of the included studies are presented in Table 2.

**Table 2:** Characteristics of included studies

Study (type of meditation)	Study design	Outcome variable	Sample size	LTMs	STMs	Controls
**Kodituwakku et al., 2012** [23] (Buddhist Insight/Vipassana meditation—(a form of mindfulness meditation)	Case–control (LTMs compared with matched controls)	**RR** (LTMs vs. control at rest) and (during meditation vs. baseline)	**23**	**13 LTMs** (4F, 9 M) Age; 25–49 years (average = 38.4 years) Meditation experience; (average 8.7 years)	**-**	**10 controls** (4F, 6 M) **demographically matched** Age; 24–49 years (average = 35.7 years) With no previous yoga or meditation experience
**Sukhsohale and Phatak, 2012** [26] (Brahmakumaris Raja Yoga meditation)	Cross-sectional (LTMs compared with STMs)	**RR** (During meditation vs. baseline)	**100** (33 M, 67 F)	**73 LTMs** Age; (mean ± SD = 53.05 ± 12.98 years) Meditation experience; (mean ± SD = 11.19 ± 5.13 years)	**27 STMs** Age; (mean ± SD = 49.37 ± 11.95 years) Meditation experience; 6 months–5 years	-
**Telles and Desiraju, 1993** [21] (Brahmakumaris Raja Yoga meditation)	Single-subject	**RR** (During meditation vs. baseline)	**18**	**18 LTMs** (All M) Age; 20–52 years (mean ± SD = 34.1 ± 8.1 years) Meditation experience; (mean ± SD = 10.1 ± 6.2 years)	-	-
**Wallance and Benson, 1972** [6] (Transcendental meditation)	Single-subject	**RR** (During meditation vs. baseline)	**36**	**36 LTMs** (28 M, 8F) Age: 17–41 years Meditation experience; (mean > 3.5 years)	-	During each test subject served as his own control (1st half-meditating, 2nd half – non-meditating)
**Lazar et al., 2005** [22] (Buddhist Insight (Vipassana) meditation—a form of mindfulness meditation)	Case–control (LTMs compared with matched controls)	**RR** (Associations with meditation practice variables)	**35**	**20 LTMs** (65% M) Age; (average = 38.2 years) Meditation experience; (average ± SD = 9.1 ± 7.1 years) and practiced; (average ± SD = 6.2 ± 4.0 h per week)	-	**15 controls**; with no meditation or yoga experience (67% M) Age; (average = 36.8 years) Matched with LTMs; by age, sex, race (all caucasians) and years of education (LTMs = 17.3 years and controls = 17.4 years)
**Wielgosz et al., 2016** [27] (Mindfulness meditation; open monitoring (OM), focused attention (FA), or loving kindness (LK) meditation)	Case–control (LTMs compared with matched controls)	**RR** (LTMs vs. control at rest)	**69**	**31 LTMs**; (17 (55%) F) Age; from 28 to 62 years (mean = 50.7 years) Meditation experience; ≥ 3 years of formal experience in mindfulness meditation	-	**38 controls** Age, sex matched meditation naive subjects; (26 (68%) F) Age; 25–65 years (mean = 47.9 years)
**Karunarathne, Amarasiri and Fernando, 2023** [24] (Budhhist meditation)	Cross-sectional comparative (LTMs compared with matched controls)	**RR, VC, TV, Spirometry parameters** (LTMs vs. control at rest)	**40**	**20 LTMs**; (11 (55%) F) Age; (mean ± SD = 45.75 ± 8.74 years) Meditation experience; (mean ± SD = 12.8 ± 6.5 years)		**20 controls** Age, sex-matched non-meditators; (11 (55%) F) Age; (mean ± SD = 45.25 ± 8.05 years)
**Telles, Nagarathna and Nagendra, 1995** [25] (“OM” meditation)	Single-subject	**RR** (During meditation vs. baseline)	**07**	**07 LTMs** (all M) Age: 29–55 years (mean ± SD = 42.3 ± 9.8 years) Meditation experience: (5–20 years)	-	**-**
**Vyas and Dikshit, 2002** [28] (Brahmakumaris Raja Yoga meditation)	Cross-sectional (LTMs & STMs compared with non-meditators)	**VC, TV**	**105**	**35 LTMs** (18 M, 17F) Age: (mean ± SD = 52.9 ± 12.4 years) Meditation experience: > 5 years	**35 STMs** (12 M, 23F) Age: (mean ± SD = 46.6 ± 13.3 years) Meditation experience: 6 months to 5 years	**35 controls** (12 M, 23F) Age: (mean ± SD = 52.6 ± 12.2 years)

*LTMs* Long-term meditators, *STMs* Short-term meditators, *M* Male, *F* Female, *RR* Respiratory rate, *SD* Standard deviation, *SE* Standard error, *VC* Vital capacity, *TV* Tidal volume, *OM* Open monitoring, *FA* Focused attention, *LK* Loving kindness

### Study participants

A total of 433 participants: 253 LTMs, 62 short-term meditators, and 118 non-meditating controls (age; ranged 17–70 years) were enrolled in the included studies. Almost all the studies recruited participants from either sex, except 2 studies [21, 25]. The included CC and CS studies compared LTMs with a matched control group of meditation-naïve participants (non-meditating controls who have never meditated), short-term meditators (STMs), or both. Single-subject (within-subject) study design was used in 3 studies [6, 21, 25], where during meditation respiratory parameters were compared with baseline resting respiratory parameters of LTMs. By the inclusion criteria, the mean practice experience of the LTMs involved in each study was ≥ 3 years, while 6 studies involved LTMs with a mean practice experience of ≥ 5 years [21–23, 25, 26, 28]. Both studies [26, 28] which included a group of STMs had recruited meditators with 6 months to 5 years’ experience in meditation as STMs. In our analysis of LTMs, we only included the participants categorized as “LTMs” in these studies, despite the possibility of having meditators ≥ 3 years in the STM group. While most of the studies recruited LTMs only based on the number of years of practice experience, 3 included studies [22, 23, 27] also considered measures of daily practice and retreat experience in addition to the total lifetime meditation practice experience in the recruitment of LTMs.

### Types of meditation techniques

Out of 9 included, 3 studies explored Brahmakumaris Raja Yoga meditation [21, 26, 28], one study [6] involved Transcendental Meditation, and another 4 studies incorporated Buddhist Vipassana/insight [22–24] and mindfulness meditation [27] while the last study was based on “OM” meditation [25].

### Quality of the evidence

The methodological quality assessment of the 3 CC studies [22, 23, 27] determined to be “good” with an overall score percentage of 80%, 70%, and 90% (Additional file 4: supplementary Table S2) respectively as assessed by the Joanna Briggs Institute (JBI) critical appraisal checklist for case–control studies [19]. Three CS studies [24, 26, 28] included in the review were determined to be “good” with all 3 rated > 75% (Additional file 5: Supplementary Table S3) as assessed by the Joanna Briggs Institute (JBI) critical appraisal checklist for analytical cross-sectional studies [19]. The Single-Case Reporting Guideline In BEhavioural Interventions (SCRIBE) 2016 checklist [20] was used to assess the methodological quality of the 3 SS-designed studies, which were rated as “fair” [6, 21, 25] scored 42.3%, 57.7%, and 57.7% respectively (Additional file 6: Supplementary Table S4).

### Summary of the findings

The included studies reported findings on respiratory function parameters of healthy LTMs including RR, vital capacity (VC), TV, and spirometry parameters and associations of these parameters with meditation practice variables.

A summary of the outcome variables assessed in each study is presented in Table 2.

### Respiratory rate

Seven out of the 9 studies included in this review reported findings related to RR in healthy LTMs. The findings on RR of LTMs were categorized under 3 sub-headings: studies that assessed the resting RR of LTMs compared to non-meditating controls, RR changes during meditation compared to the resting baseline, and associations of RR with meditation practice variables of LTMs.

#### Resting respiratory rate

**Fig. 2:**
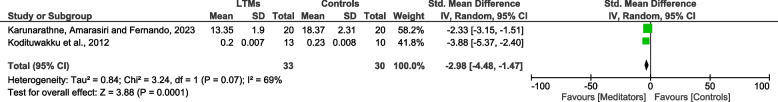
Resting RR among LTMs compared to non-meditating controls

Resting RR in healthy LTMs compared to matched non-meditating controls was assessed in 3 studies [23, 24, 27]. The summary of the effect sizes and 95% confidence interval (CI) of the 2 studies [23, 24] which reported significant differences and the mean ± SD values for both meditator and control groups are shown in Fig. 2. The pooled results of the 2 studies [23, 24] reported a standard mean difference (SMD) of − 2.98 in resting RR between LTMs and non-meditating controls, with a 95% CI ranging from − 4.48 to − 1.47. The overall *z*-score was 3.88 (*p* < 0.001) and a moderate heterogeneity (*I*^2^ = 69%) was reported among the 2 individual studies.

The third study on resting RR of LTMs [27] which did not report the exact mean ± SD values for the study groups also showed a similar trend towards slower RR in LTMs compared to non-meditating controls with a nearly-significant mean difference (MD) of − 1.6 breaths/minute (*p* = 0.053).

#### During meditation vs. baseline

Five studies assessed RR during meditation compared to the baseline RR in LTMs. Out of the 5 studies, 2 [23, 26] reported significantly lower RR during meditation compared to baseline, and presented mean ± SD values at multiple time intervals during meditation. Another study [6] reported decreased RR during meditation by 2 breaths/minute with no data on the significance of this difference compared to baseline or the exact mean and SD values for each phase. The other 2 studies [21, 25] reported no significant difference in RR during meditation compared to the respective baseline RRs. Due to the heterogeneity of data presentation in the above studies, pooling of data was not feasible and hence effect estimates and the 95% CI values were reported separately for individual studies (Table 3).

**Table 3:** Studies assessing RR during meditation compared to baseline in LTMs

Study	RR measured at	Study findings	Mean/SD, effect estimates and confidence intervals
Sukhsohale and Phatak, 2012 [26]	Baseline, during meditation at 2-time points (at 15 minutes of meditation and 30 minutes of meditation)	RR significantly decreased during meditation both at 15 minutes and 30 minutes of meditation compared to baseline.	a). RR at 15 minutes of meditation vs. baseline; [SMD= −0.4, overall effect= 2.41 (*p*= 0.02), 95% CI (−0.73 to −0.08)] b). RR at 30 minutes of meditation vs. baseline; [SMD= −0.5, overall effect = 2.97 (*p*=0.003), 95% CI (−0.83 to −0.17)]
Kodituwakku et al., 2012 [23]	Baseline, during meditation at 3 time points (early, middle, and late; each 8 minutes)	RR decreased during meditation at all 3 time intervals (early, middle, late) compared to the baseline reporting the highest level of significance in the early phase (*p*<0.001).	a). RR at early phase of meditation vs. baseline; [SMD= −7.19, overall effect= 6.25 (*p*<0.00001), 95% CI (−9.45 to −4.94)] b). RR at middle phase of meditation vs. baseline; [SMD= −0.51, overall effect= 1.28 (*p*=0.2), 95% CI (−1.29 to 0.27)] c). RR at late phase of meditation vs. baseline; [SMD=−0.48, overall effect= 1.2 (*p*=0.23), 95% CI (−1.26 to 0.3)]
Wallance and Benson, 1972 [6]	Baseline, during meditation	RR decreased during meditation compared to the baseline.	MD= 2 breaths/minute; (mean/SD data, level of significance not reported)
Telles, Nagarathna and Nagendra, 1995 [25]	Baseline, during meditation	No significant difference in RR during meditation compared to baseline.	a). RR during meditation = (mean ±SD, 10.4 ±3.3), b). Baseline RR = (mean ±SD, 10.8 ±3.6); [MD= −0.4, overall effect =0.22 (*p*= 0.83), 95% CI = 3.22]
Telles and Desiraju, 1993 [21]	Baseline, during meditation	No significant difference in RR during meditation compared to the proceeding baseline.	Pre-meditation RR= (mean ±SD; 12.1 +2.4 breaths/40s), during meditation period-with target thinking; = (mean ±SD; 13.4 +3.5 breaths/40s) [MD= 1.3, overall effect =1.3 (*p*= 0.19), 95% CI = 3.26]

*MD* Mean difference, *SMD* Standard mean difference, *SD* Standard deviation, *95% CI* Confidence interval at 95%

#### Associations with meditation practice variables

Three studies included in this review reported significant associations between RR and meditation practice variables (Table 4).

**Table 4:** Studies reported on associations between RR and meditation practice variables

Study	Findings
Lazar et al., 2005 [22]	Mean RR change during meditation from baseline (mean RR from 6 minute baseline – mean RR during first 6 minutes of meditation) significantly correlated with both, the total number of years of meditation practice (*r*=−0.57, *p*= 0.009) and the self-reported total number of hours of formal sitting meditation over the lifetime (*r*=−0.75, *p* <0.001) of LTMs.
Wielgosz et al., 2016 [27]	Greater practice experience of the LTMs was associated with slower baseline respiration, independently of age and gender and this association was specific to intensive retreat experience and was not associated with daily routine practice duration. A significant inverse relationship was observed between resting RR in LTMs and retreat hours. A doubling of the retreat hours was associated with a decrease in RR by 0.7 breaths/minute, 95% CI (0.07, 1.33), *p*=0.032.
Karunarathne, Amarasiri and Fernando, 2023 [24]	The resting RR of LTMs showed a significant negative correlation with uninterrupted, continuous total lifetime meditation practice in years (*r* =−0.444, *p* =0.049), and the average length of a meditation session per day (*r* =−0.65, *p* =0.002). The LTMs with a longer duration of retreat participation experience had slower resting RR (*r* =−0.522, *p* =0.018) and higher tidal volumes (*r* = 0.474, *p* =0.04).

### Spirometry parameters, lung volumes, and lung capacities

**Fig. 3:**
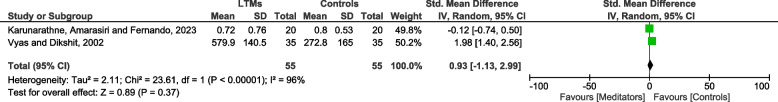
Tidal volume of LTMs compared to non-meditating controls

**Fig. 4:**
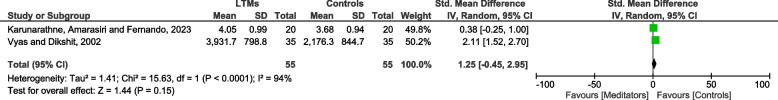
Vital capacity of LTMs compared to non-meditating controls

Out of the 9 included studies, 2 studies [24, 28] looked at spirometry parameters, lung volumes, and lung capacities. Both studies [24, 28] assessed TV, VC, and spirometry, and the effect estimates for these outcome variables are presented in Figs. 3 and 4 respectively.

The SMD in TV between LTMs and non-meditating controls was 0.93, with a 95% CI ranging from − 1.13 to 2.99. The overall effect score (*z*) was 0.89 (*p* = 0.37). A high level of heterogeneity was noted among the included studies with an *I*^2^ value of 96%.

The non-significant overall effect *z*-score for VC was 1.44 (*p* = 0.15), while SMD between LTMs and controls was 1.25 with a 95% CI ranging from − 0.45 to 2.95.

The effect estimates for other spirometry parameters in LTMs compared to non-meditating controls as reported in a single study [24] are shown in Table 5.

**Table 5:** Effect estimates for spirometry parameters

Outcome variable	Findings	MD (95% CI)	Overall effect; *z*-score (*p* value)
**FVC (l)**	LTMs; mean ±SD= (3.81 ±0.94), NMs; mean ±SD= (3.47 ±0.93)	0.34 (−0.24, 0.92)	1.15 (*p*= 0.25)
**FEV**_**1**_** (l)**	LTMs; mean ±SD= (3.16 ±0.84), NMs; mean ±SD= (2.95 ±0.72)	0.21 (−0.27, 0.69)	0.85 (*p*= 0.4)
**PEFR (l/s)**	LTMs; mean ±SD= (9.89 ±2.49), NMs; mean ±SD= (8.22 ±2.28)	1.67 (0.19, 3.15)	2.21 (*p*= 0.03)

## Discussion

In this review, we summarized the available evidence on selected respiratory function parameters of healthy LTMs. Findings revealed that LTMs appear to have slower baseline RR compared to non-meditating controls and lower RR during meditation compared to baseline, and both these outcome variables appear to be significantly associated with some meditation practice variables (meditation practice experience in years/ hours, intensive retreat participation experience). Evidence on spirometry parameters, lung volumes, and lung capacities was limited.

### Resting RR

All 3 independent studies which explored the resting RR in LTMs reported a consistent trend towards a slower resting RR in LTMs compared to their matched non-meditating controls. The meta-analysis of the 2 studies [23, 24] demonstrated a highly significant substantial effect size (3.88) with an SMD of − 2.98. The 95% CI was entirely below zero, suggesting a consistent effect across the studies with all values favoring the LTM group. The findings of the study by Wielgosz and the team [27] also aligned with the trend observed in the above 2 studies reporting a nearly significant (*p* = 0.053) result of slower RR among LTMs (MD = − 1.6 breaths/minute) compared to the non-meditators. Overall findings of the 3 included studies indicated a clear and robust effect of long-term practice of meditation towards establishing a slower baseline RR in LTMs. While emphasizing the strength of the observed effect, it is important to acknowledge the moderate heterogeneity (69%) reported among the studies included in the analysis which could be a result of variations in participant characteristics, meditation techniques practiced, and measurement methods used in the included studies. This presence of heterogeneity among the studies, the low number of studies employed in the analysis (< 10), and the nearly significant results of one study should be acknowledged as limitations that could impact the generalizability of the results. Our findings prompt future research to elucidate the underlying mechanisms and clinical implications of this observed slower resting respiration in LTMs.

### During meditation vs. baseline RR

A slower RR during meditation practice relative to the baseline is expected in most of the meditation practices [22]. Though 3 out of the 5 studies assessing RR changes during meditation compared to baseline reported slower RR during meditation, these studies reflected a higher diversity in regard to research methodologies and data presentation. The 2 included studies [23, 26] provided strong evidence of significantly slower RR during meditation compared to baseline in LTMs. The study [26] by Sulokshana et al. (2012) reported a consistent, statistically significant reduction in RR during meditation according to the presented data at 15 min and 30 min of meditation (Table 3). Findings of the study [23] by Kodithuwakku et al. (2012) reported a remarkable decrease in RR during the early 8-min period of meditation with an overall effect of 6.25 (*p* < 0.001). However, the decrease in RR compared to baseline in middle- and late-8-min periods of meditation did not meet the statistical significance (Table 3). While findings of both of these studies underscored the immediate reductions in RR during meditation in LTMs, variations in significance across different time intervals during meditation as observed in the study by Kodithuwakku et al. (2012) raise the importance of exploring the temporal dynamics of RR changes during meditation.

The study [6] by Wallance and Benson also reported a reduction in RR during meditation by 2 breaths/minute though the significance of this change was not documented. More detailed data presentation in meditation studies should be encouraged to facilitate more robust analyses leading to a better understanding of RR changes during meditation.

The other 2 studies [21, 25] reported no significant difference in RR during meditation compared to baseline. A relatively small sample size (*n* = 18 and *n* = 7 respectively) involved in these studies compared to other studies could have potentially limited the statistical power leading to the non-significant findings of these studies. Also, the latter 3 studies [6, 21, 25] were rated “fair” in the risk of bias assessment falling within the (34–66%) quality rating range according to the SCRIBE 2016 checklist (Additional file 6) indicating certain limitations which could have affected the precision and reliability of their findings. This review emphasizes the importance of future research with adequate samples and higher methodological quality to confirm and expand the reported results in this regard.

From a physiological standpoint, a dramatic reduction in RR during meditation indicates the activation of an integrated hypothalamic response, which is recently called “the relaxation response” [6, 29]. This response appears to be associated with decreased sympathetic nervous system activity which is hypothesized to be the counterpart of the “fight or flight” response [6]. Therefore, reduced RR during meditation could be a representation of the heightened activation of the parasympathetic nervous system and downregulated sympathetic activity promoting a relaxed, stress-free state. Though increased parasympathetic function is expected during most meditation techniques, exceptions are not rare [30] calling out more investigations on physiological changes during meditation concerning specific types of meditation techniques to understand intrinsic characteristics and mechanisms bound with different meditation techniques.

The typical RR in humans falls within the range of 12–20 breaths per minute (BPM). Controlled slower respiration at 6 BPM was found to be associated with increased venous return [12], a marked reduction in blood pressure (BP) [11], and was found to be optimal for increasing arterial oxygenation in healthy humans by improving alveolar ventilation and reducing dead space leading to an increased ventilation-perfusion ratio [31]. In this context, it appears that meditation involving breathing at slower RR seems to be beneficial by improving gas exchange and reducing cardiac work by reducing BP. Also, respiration at slower rates was found to be associated with increased vagal activation and a shift of the autonomic balance towards parasympathetic dominance by modulating autonomic-cardiovascular regulation [13, 14]. Respiration is a powerful modulator of heart-rate variability (HRV) [32] where controlled respiration at slower rates appears to be effective in preserving autonomic function and maximizing HRV, possibly contributing to decreased morbidity and increased longevity in healthy individuals [11].

### Associations with meditation practice variables

A group of researchers [22] investigating the experience-dependent cortical plasticity and cortical thickness associated with measures of meditation practice experience in LTMs assessed the associations of RR in LTMs with some selected measures of their meditation practice experience. They tested whether the changes in RR between rest and during meditation could serve as an objective measure of meditation practice experience and they found significant correlations for mean RR change during meditation from baseline with both, the total number of years of meditation practice, and the self-reported total number of hours of formal sitting meditation over the lifetime in LTMs. Based on these observations and the correlations between RR change and cortical thickness, authors concluded that changes in RR during meditation compared to baseline could be considered as a physiological measure of cumulative meditation practice experience.

Wielgosz and the team [27] who observed that the baseline RR of LTMs practicing mindfulness meditation was significantly slower than the baseline RR of their matched controls further attempted to establish an association between greater practice experience and slower baseline RR. They recorded RR in LTMs during uninstructed rest and at 3 separate experimental sessions, spaced on average 4.5 months apart. Across the 3 sessions, they observed a strong inverse relationship between total lifetime meditation practice experience (in hours) and basal RR among LTMs. This observation was further strengthened by the recent work [24], which reported a significant negative correlation of resting RR of LTMs with total cumulative lifetime meditation practice experience (in years). Therefore, greater practice experience of LTMs appears to be associated with slower RR in LTMs during uninstructed rest [24, 27], where greater practice amplifies the effect.

Furthermore, consistent findings on associations of both baseline RR and mean RR change during meditation from baseline with lifetime meditation practice experience provide insights into which changes in RR caused by effortful meditation practice could generalize to habitual characteristics of the meditators over time. Longitudinal studies of LTMs, exploring the changes in respiratory function parameters at multiple time intervals, would be of great value to further elucidate how these observed direct effects of sustained mediation practice would generalize to habitual characteristics.

It is important to note that the association between slower baseline RR in LTMs and greater practice experience was specific to intensive retreat practice experience [24, 27]. This could be explained by the fact that intensive retreats provide physical space and a supportive environment for engagement in deep meditation practice for longer durations, minimizing the possible distractions and obligations of daily life. The effects of intensive retreat meditation practice are an understudied area of interventional meditation research in the empirical literature. To fill this knowledge gap, the authors recommend considering intensive retreat meditation training as an important element in future experimental studies in meditation research, where information on retreat practice experience in observational studies would strengthen the current evidence.

### Spirometry parameters, lung volumes, and lung capacities

Data on spirometry parameters, lung volumes, and lung capacities are crucial in assessing the respiratory function of healthy individuals. Two studies [24, 28], included in this study, assessed the 2 important static lung volumes/capacities, TV and VC, while only one recent study [24] reported on spirometry parameters of healthy LTMs. Findings of both TV (SDM = 0.93) and VC (SDM = 1.25) indicated a non-significant difference between LTMs and matched non-meditating controls reporting an overall effect *z*-score of 0.89 (*p* = 0.37) and 1.44 (*p* = 0.15) respectively. A high level of heterogeneity was reported for the findings on TV and VC of LTMs, reflecting high variability among the included studies pertaining to the differences between the 2 studies in regards to the participant characteristics, meditation techniques, and measurement standards.

Considering the findings related to other spirometry parameters, both FVC and FEV_1_ parameters did not differ between LTMs and matched non-meditators. In contrast, the PEFR in LTMs was significantly higher than their matched controls. Further investigations are needed to ascertain the clinical significance of these differences in the context of healthy individuals and those with compromised respiratory function. We acknowledge that these findings are based on a single study and hence further research with larger samples assessing spirometry parameters in standard methods is recommended to extend these observations and their clinical significance. Future studies with longitudinal designs and consistent measurement techniques would aid in understanding the possible beneficial changes in these respiratory parameters with the long-term practice of meditation over time. Considering the beneficial effects of controlled slow respiration and slower respiration is expected in most meditative practices, exploring respiratory function in LTMs would offer potential insight into the pathways by which contemplative/meditative practice may lead to this wide array of beneficial physiological changes. Discovery of whether such meditation practices are capable of positively influencing respiratory function would be useful to develop meditation-based clinical interventions. Further comprehensive research is recommended to discover the associations between long-term meditation and respiratory function in relation to the effect modifiers/specifics of meditation (type of meditation technique, total lifetime practice experience, retreat experience, consistency of practice, etc.) and population characteristics (age, gender, and ethnicity).

### Limitations and strengths

Limitations of the included studies were inconsistent reporting of the nature of participants’ practice experience, and great variety in reporting results, smaller sample size, and average methodological quality.

Limitations of the review include missing any evidence of research interest published in other languages except for English, and due to extensive heterogeneity of interventions, comparison groups, and reporting outcomes, conducting a complete meta-analysis was infeasible to draw strong conclusions. We acknowledge that the varying definitions of “LTMs” among the included studies introduced heterogeneity into our analysis. For example, in regard to the 2 studies that included a group of STMs whose practice experience ranged from 6 months to 5 years, there may have been participants with ≥ 3 years of experience in the STM group. However, those were not considered as LTMs in our analysis and we only included the participants who were categorized as “LTMs” in the respective studies.

The comprehensive search strategy, selection of studies by two independent reviewers, methodological quality assessed for all included studies, and detailed summary of available evidence with extensive discussion on the topic are the strengths of this study.

## Conclusions

Long-term meditation appears to be associated with slower baseline respiration in healthy individuals, with a trend of immediate reduction in RR during meditation in LTMs. The baseline RR and its changes during meditation in LTMs could be considered a physiological measure of their cumulative meditation practice experience. Standardized assessment of spirometry parameters, lung volumes, and lung capacities in LTMs and their associations with meditation practice variables are limited and warrant further research. Whether these observed respiratory function changes caused by effortful meditation practice could be generalized to habitual characteristics of healthy individuals over time will be an important focus of future studies.

### Supplementary Information

**Additional file 1: Supplementary Table S1.** PRISMA 2020 Checklist: Respiratory function in healthy long-term meditators: A systematic review.

**Additional file 2.**

**Additional file 3.** Search strategy.

**Additional file 4: Supplementary Table S2.** Quality Assessment of the Case-Control Studies.

**Additional file 5: Supplementary Table S3.** Quality Assessment Tool for Cross-Sectional Studies.

**Additional file 6: Supplementary Table S4.** Quality Assessment Tool for Studies of Single Subject Study Design.

## Data Availability

All data generated or analyzed during this study are included in this published article (and its supplementary information files). The protocol (Additional file 2) of this review was not registered elsewhere.

## Abbreviations

LTMs: Long-term meditators
STMs: Short-term meditators
CC: Case-control
CS: Cross-sectional
SS: Single-subject
JBI: Joanna Briggs Institute
SCRIBE: Single-Case Reporting Guideline In BEhavioural Interventions Statement
RR: Respiratory rate
TV: Tidal volume
VC: Vital capacity
FEV_1_: Forced expiratory volume in 1 s
FVC: Forced vital capacity
BPM: Breaths per minute
BP: Blood pressure
HRV: Heart-rate variability
