# Supplementary material for: Respiratory function in healthy long-term meditators: a systematic review
Source: Syst Rev. 2024 Jan 2;13:1. doi: 10.1186/s13643-023-02412-0 (PMC10759765; doi:10.1186/s13643-023-02412-0)
Supplement: Supplementary file 2 — Additional file 2. [file 13643_2023_2412_MOESM2_ESM.docx]

**Additional file 2**

**Respiratory function in healthy long-term meditators: Protocol for a systematic review**

L.J.U. Karunarathne^1^, W.A.D.L. Amarasiri^2^, A.D.A. Fernando^2^

**Review Question:**

To explore selected respiratory function parameters (RR, lung volumes, lung capacities, and spirometry parameters) in healthy adult long-term meditators and their associations with meditation practice variables.

**Searches:**

- **Sources:** **PubMed, MEDLINE, Cochrane Library (CENTRAL), Google Scholar** [Revision: Updated to include searches from **PubMed, EMBASE (Ovid), Scopus, Proquest Dissertation and Thesis Global databases, CENTRAL,** and **Google Scholar**]
- **Search dates:** From the first available date to 30th October 2022 [Revision: Updated to include searches **from the year 1950 until August 15th, 2023**]
- **Language:** English language
- **Keywords:** “Meditation”, “long-term meditation”, “long-term meditators”, “healthy” AND keywords related to respiratory function (“respiratory function” OR “pulmonary function” OR “lung function” OR spirometry).

**Type of studies to be included:** Controlled trials, and observational studies (cross-sectional, longitudinal, case-control, and cohort)

**Condition or domain being studied:**

The selected respiratory function parameters (RR, lung volumes, lung capacities, and spirometry parameters) at rest and during meditation in healthy long-term meditators.

**Participants/ population:**

Healthy, adult long-term meditators (LTMs) practicing any meditation technique denoted by the umbrella term “meditation” will be included in this study [A meditator with a mean practice experience of at least 3 years or more in a particular type of meditation, regardless of the daily routine practice and retreat experience was defined as a “long-term meditator” to select studies to be included in this review].

Studies involving unhealthy/diseased individuals, monks as “LTMs” and those younger than 16 years or older than 70 years were excluded.

**Intervention (s)/ exposure (s):**

Long-term meditation (regular meditation practice for at least 3 years or more) is the intervention or the exposure of this review.

**Comparator (s)/ control:**

Studies comparing selected respiratory function parameters at rest in healthy LTMs with meditation-naïve participants (non-meditators with no experience in meditation), short-term meditators, or both, and/or studies comparing selected respiratory function parameters of LTMs during meditation with the same respiratory parameters measured at rest.

**Main Outcome (s):**

The primary objective of this review will be to assess selected respiratory function parameters (RR, lung volumes, lung capacities, and spirometry parameters) at rest in healthy adult LTMs with >3 years of meditation experience, compared to meditation-naïve (non-meditating) participants.

This review will also summarize selected respiratory function changes during meditation compared to baseline rest in healthy adult LTMs, to identify immediate responses during the practice of meditation in healthy LTMs and will explore the influence of various meditation practice variables (e.g., cumulative meditation practice experience, total hours of sitting meditation, etc.) on selected respiratory function parameters in healthy LTMs.

**Additional outcome (s):**

To identify the associations of various meditation practice variables (e.g., cumulative meditation practice experience, total hours of sitting meditation, etc.) with selected respiratory function parameters in healthy LTMs.

**Data extraction (Selection and coding):**

Study selection will be done by two independent reviewers and any disagreement will be resolved by discussion with a third reviewer.

Data will be extracted by two independent reviewers using a pre-designed data collection form. Any disagreements will be discussed with the third reviewer. All the data extracted will be recorded in an Excel sheet.

**Risk of bias (Quality) assessment:**

All the included studies will be assessed for methodological rigor by two independent reviewers. Any discrepancies will be resolved by discussion with the third author. The methodological quality of the randomized controlled trials (RCTs) will be assessed using the Cochrane risk of bias tool and the risk will be assessed across the following domains: random sequence generation, allocation concealment, blinding of participants and personnel (checking for possible performance bias), blinding of outcome assessment checking for possible detection bias), incomplete outcome data, selective reporting, and other biases. Three risks of bias categories for RCTs i.e. low, high, and unclear will be set for each domain. Accordingly, overall bias will be set for each study as high (if all categories were met), moderate (if two or more criteria were only partially met), and low (if all criteria were not met or only one criterion was met).

For the quality assessment of the cross-sectional and case-control studies, the Joanna Briggs Institute (JBI) Critical Appraisal Tools will be used. Single-subject designed studies will be assessed against the Single-Case Reporting Guideline In BEhavioural Interventions (SCRIBE) Statement. In both of these quality assessment tools of the observational studies, each component will be evaluated as “Yes”, “No”, or “Other (unclear/ not applicable)” and will be rated as (“Yes”=1, “No”=0, “other”= 0). An overall rating will be provided for each study. Accordingly, the quality score will be determined by the range 67–100 (good), 34–66 (fair), and 0–33 (bad).

**Strategy for data synthesis:**

Collected data will be synthesized with the use of Review Manager (RevMan) software version 5.4.1. The analyses will be complemented by computing effect estimates for selected respiratory function parameters. Data will be summarized for each outcome variable with standardized mean difference (SMD) and mean difference (MD) where appropriate presenting 95% confidence interval (CI) ranges, overall effect size, and its significance level for each. A random-effects model will be used to pool the data. Statistical heterogeneity across studies will be tested using I² (I²) static [I² <25% indicates low, I² = 26-74% moderate, and I² >75% indicates high heterogeneity]. Statistical significance will be defined as p-value <0.05. Findings from the observational studies where quantitative analysis is not possible will be synthesized narratively where appropriate. [Updated]

**Contact details for further information:**

L.J.U. Karunarathne ([rhdc201914@stu.cmb.ac.lk/](mailto:rhdc201914@stu.cmb.ac.lk/) [udanikarunarathne90@gmail.com](mailto:udanikarunarathne90@gmail.com))

**Organizational affiliations of the review:** None

**Review team members and their organizational affiliations:**

L.J.U. Karunarathne, Department of Physical Medicine, National Hospital of Colombo, Sri Lanka.

W.A.D.L. Amarasiri, Department of Physiology, Faculty of Medicine, University of Colombo, Sri Lanka.

A.D.A. Fernando, Department of Physiology, Faculty of Medicine, University of Colombo, Sri Lanka.

**Collaborators:**

Miss H.M.N.N. Dasanayaka, Research Promotion and Facilitation Center, Faculty of Medicine, University of Colombo, Sri Lanka

Dr. Yasith Mathagaweera, Lecturer, Department of Anatomy, Faculty of Medicine, University of Colombo, Sri Lanka.

**Type and method of review:** Systematic review

**Anticipated or actual start date:** October 2021

**Anticipated completion date:** October 2022

**Funding sources/sponsors:** No funding

**Conflicts of interest:** None

**Language:** English language

**Countries involved:**

This systematic review will be carried out by a group of authors based in Sri Lanka.

**Stage of review:** Review completed, not yet published

**Date of first protocol development:** August 2021

**Date of revisions made:** August 2023

**Revision notes:** The search was updated adding new search sources. The strategies for data synthesis were updated.
