# Supplementary material for: Respiratory function in healthy long-term meditators: a systematic review
Source: Syst Rev. 2024 Jan 2;13:1. doi: 10.1186/s13643-023-02412-0 (PMC10759765; doi:10.1186/s13643-023-02412-0)
Supplement: Supplementary file 3 — Additional file 3. Search strategy. [file 13643_2023_2412_MOESM3_ESM.docx]

**Additional file 3: Search strategy**:

*Respiratory function in healthy long-term meditators: A systematic review*

Search strategies with specific search terms used for each database.

1. **PubMed:**

*Search restricted to studies published from 1950 – 2023/08/15; with “English” and “humans” filters

|  | Search terms | No. of results |
| --- | --- | --- |
| *Search Query:* | (meditation) AND ((Respiratory function OR pulmonary function OR lung function OR Spirometry)) AND (english[Filter]) AND (humans[Filter]) AND (1950:2023/08/15 [pdat]) AND (english[Filter]) Filters: English | 298 |
| *Complete search strategy:* | (("meditate"[All Fields] OR "meditated"[All Fields] OR "meditating"[All Fields] OR "meditation"[MeSH Terms] OR "meditation"[All Fields] OR "meditations"[All Fields] OR "meditation s"[All Fields] OR "meditational"[All Fields] OR "meditative"[All Fields] OR "meditator"[All Fields] OR "meditators"[All Fields]) AND ("respiratory physiological phenomena"[MeSH Terms] OR ("respiratory"[All Fields] AND "physiological"[All Fields] AND "phenomena"[All Fields]) OR "respiratory physiological phenomena"[All Fields] OR ("respiratory"[All Fields] AND "function"[All Fields]) OR "respiratory function"[All Fields] OR "respiration"[MeSH Terms] OR "respiration"[All Fields] OR (("lung"[MeSH Terms] OR "lung"[All Fields] OR "pulmonary"[All Fields]) AND ("functional"[All Fields] OR "functional s"[All Fields] OR "functionalities"[All Fields] OR "functionality"[All Fields] OR "functionalization"[All Fields] OR "functionalizations"[All Fields] OR "functionalize"[All Fields] OR "functionalized"[All Fields] OR "functionalizes"[All Fields] OR "functionalizing"[All Fields] OR "functionally"[All Fields] OR "functionals"[All Fields] OR "functioned"[All Fields] OR "functioning"[All Fields] OR "functionings"[All Fields] OR "functions"[All Fields] OR "physiology"[MeSH Subheading] OR "physiology"[All Fields] OR "function"[All Fields] OR "physiology"[MeSH Terms])) OR ("respiratory physiological phenomena"[MeSH Terms] OR ("respiratory"[All Fields] AND "physiological"[All Fields] AND "phenomena"[All Fields]) OR "respiratory physiological phenomena"[All Fields] OR ("lung"[All Fields] AND "function"[All Fields]) OR "lung function"[All Fields]) OR ("spirometry"[MeSH Terms] OR "spirometry"[All Fields] OR "spirometries"[All Fields])) AND "english"[Language] AND "humans"[MeSH Terms] AND 1950/01/01: 2023/08/15 [Date - Publication] AND "english"[Language]) AND (english[Filter])  **Translations**  **meditation:** "meditate"[All Fields] OR "meditated"[All Fields] OR "meditating"[All Fields] OR "meditation"[MeSH Terms] OR "meditation"[All Fields] OR "meditations"[All Fields] OR "meditation's"[All Fields] OR "meditational"[All Fields] OR "meditative"[All Fields] OR "meditator"[All Fields] OR "meditators"[All Fields]  **Respiratory function:** "respiratory physiological phenomena"[MeSH Terms] OR ("respiratory"[All Fields] AND "physiological"[All Fields] AND "phenomena"[All Fields]) OR "respiratory physiological phenomena"[All Fields] OR ("respiratory"[All Fields] AND "function"[All Fields]) OR "respiratory function"[All Fields] OR "respiration"[MeSH Terms] OR "respiration"[All Fields]  **pulmonary:** "lung"[MeSH Terms] OR "lung"[All Fields] OR "pulmonary"[All Fields]  **function:** "functional"[All Fields] OR "functional's"[All Fields] OR "functionalities"[All Fields] OR "functionality"[All Fields] OR "functionalization"[All Fields] OR "functionalizations"[All Fields] OR "functionalize"[All Fields] OR "functionalized"[All Fields] OR "functionalizes"[All Fields] OR "functionalizing"[All Fields] OR "functionally"[All Fields] OR "functionals"[All Fields] OR "functioned"[All Fields] OR "functioning"[All Fields] OR "functionings"[All Fields] OR "functions"[All Fields] OR "physiology"[Subheading] OR "physiology"[All Fields] OR "function"[All Fields] OR "physiology"[MeSH Terms]  **lung function:** "respiratory physiological phenomena"[MeSH Terms] OR ("respiratory"[All Fields] AND "physiological"[All Fields] AND "phenomena"[All Fields]) OR "respiratory physiological phenomena"[All Fields] OR ("lung"[All Fields] AND "function"[All Fields]) OR "lung function"[All Fields]  **Spirometry:** "spirometry"[MeSH Terms] OR "spirometry"[All Fields] OR "spirometries"[All Fields]  **english[Filter]:** english [LA]  **humans[Filter]:** humans[MH]  **english[Filter]:** english [LA] |  |

1. **Scopus:**

*Search restricted to studies published till 2023/August; with “English” and “humans” filters

|  | Search terms | No. of results |
| --- | --- | --- |
| *Search Query:* | ( meditation* ) AND ( respiratory AND function* OR lung AND function* OR spirometry* ) AND ( healthy OR control ) AND ( LIMIT-TO ( DOCTYPE , "ar" ) ) AND ( LIMIT-TO ( LANGUAGE , "English" ) ) | 2, 192 |

1. **EMBASE (Ovid):**

*Search restricted to studies published from 1950 – 2023/August; with “English” and “humans” filters

|  | Search terms | No. of results |
| --- | --- | --- |
| *Search Query:* | *meditation/ AND (lung function/ OR respiratory function/ OR spirometry/)  Limit = English Language | 47 |

1. **Cochrane Central Register of Controlled Trials (CENTRAL)**

*Search restricted to studies published from 1950/January – 2023/August

|  | Search terms | No. of results |
| --- | --- | --- |
| *Search Query #1* | (Meditation OR "long-term meditation" OR "long-term meditators") | 4, 505 |
| *Search Query #1* | ("respiratory function" OR "Pulmonary function" OR "lung function" OR spirometry) | 29, 093 |
| *Search Query #3 (#1 + #2)* | (*(Meditation OR "long-term meditation" OR "long-term meditators") AND ("respiratory function" OR "Pulmonary function" OR "lung function" OR spirometry)) (Word variations have been searched)" with Publication Year from 1950 to 2023, with Cochrane Library publication date Between Jan 1950 and Aug 2023, in Trials (Word variations have been searched) | 47 |

1. **Grey literature search**

|  | Search terms | No. of results |
| --- | --- | --- |
| *Google Scholar* | ("long-term meditation" OR "long-term meditator") AND ("Respiratory function" OR "pulmonary function" OR "lung function" OR "spirometry") AND "healthy"  *Search restricted to studies published from 1950 – 2023, and Published in “English” language | 85 |
| *ProQuest Dissertations & Theses Global* | Long-term meditation AND (Respiratory function OR Pulmonary function OR Lung function OR Spirometry)  Filters:   - Dissertations & Theses OR Scholarly Journals - 1950 - 2023-08-15 | 4,651 |
