# Supplementary material for: Respiratory function in healthy long-term meditators: a systematic review
Source: Syst Rev. 2024 Jan 2;13:1. doi: 10.1186/s13643-023-02412-0 (PMC10759765; doi:10.1186/s13643-023-02412-0)
Supplement: Supplementary file 4 — Additional file 4: Supplementary Table S2. Quality Assessment of the Case-Control Studies. [file 13643_2023_2412_MOESM4_ESM.docx]

**Additional file 4: Supplementary Table S2:**

**Quality Assessment of the Case-Control Studies**

| **Joanna Briggs Institute (JBI) critical appraisal checklist for case-control studies** | | | | | | | | | | | | | |
| --- | --- | --- | --- | --- | --- | --- | --- | --- | --- | --- | --- | --- | --- |
| **Study** | **1** | **2** | **3** | **4** | **5** | **6** | **7** | **8** | **9** | **10** | **Total score*** | **Quality rating** | **Include/**  **Exclude** |
| (Kodituwakku *et al*., 2012) | **Y** | **Y** | **Y** | **Y** | **Y** | U | U | **Y** | NA | **Y** | 7/10 | (70%)  Good | Included |
| (Lazar *et al*., 2005) | **Y** | **Y** | **Y** | **Y** | **Y** | **Y** | U | **Y** | NA | **Y** | 8/10 | (80%)  Good | Included |
| (Wielgosz *et al*., 2016) | **Y** | **Y** | **Y** | **Y** | **Y** | **Y** | **Y** | **Y** | NA | **Y** | 9/10 | (90%)  Good | Included |

*****Score gained and the percentage of maximum score based on JBI appraisal for case-control studies (10 criteria); Moola S, Munn Z, Tufanaru C, Aromataris E, Sears K, Sfetcu R, Currie M, Qureshi R, Mattis P, Lisy K, Mu P-F. Chapter 7: Systematic reviews of etiology and risk. In: Aromataris E, Munn Z (Editors). JBI Manual for Evidence Synthesis. JBI, 2020. Available from <https://synthesismanual.jbi.global>

***Answers:*** **Y=YES,** N**=NO,** U **=UNCLEAR,** NA**=NOT APPLICABLE**

***Total score:*** Number of YES; (“YES”=1, “NO”=0, “Other: (U/ NA)” = 0) and calculated the percentage of the total score

***The quality rating:*** 67-100 **(Good)**, 34-66 **(Fair)**, and 0-33 **(Poor)**.

**Questions (10 criteria):**

**1**. Were the groups comparable other than the presence of disease in cases or the absence of disease in controls?

**2.** Were cases and controls matched appropriately?

**3.** Were the same criteria used for identification of cases and controls?

**4**. Was exposure measured in a standard, valid and reliable way?

**5.** Was exposure measured in the same way for cases and controls?

**6**. Were confounding factors identified?

**7.** Were strategies to deal with confounding factors stated?

**8**. Were outcomes assessed in a standard, valid and reliable way for cases and controls?

**9.** Was the exposure period of interest long enough to be meaningful?

**10**. Was appropriate statistical analysis used?
