# Supplementary material for: Respiratory function in healthy long-term meditators: a systematic review
Source: Syst Rev. 2024 Jan 2;13:1. doi: 10.1186/s13643-023-02412-0 (PMC10759765; doi:10.1186/s13643-023-02412-0)
Supplement: Supplementary file 5 — Additional file 5: Supplementary Table S3. Quality Assessment Tool for Cross-Sectional Studies. [file 13643_2023_2412_MOESM5_ESM.docx]

**Additional file 5: Supplementary Table S3:**

**Quality Assessment Tool for Cross-Sectional Studies**

| **Joanna Briggs Institute (JBI) critical appraisal checklist for analytical cross-sectional studies** | | | | | | | | | | | |
| --- | --- | --- | --- | --- | --- | --- | --- | --- | --- | --- | --- |
| **Study** | **1** | **2** | **3** | **4** | **5** | **6** | **7** | **8** | **Total score*** | **Quality rating** | **Include/**  **Exclude** |
| Sukhsohale and Phatak, 2012 | **Y** | **Y** | **Y** | **Y** | **U** | **N** | **Y** | **Y** | 6/8 | (75%)  Good | Included |
| Vyas and Dikshit, 2002 | **Y** | **Y** | **Y** | **Y** | **U** | **U** | **Y** | **Y** | 6/8 | (75%)  Good | Included |
| Karunarathne, Amarasiri and Fernando, 2023 | **Y** | **Y** | **Y** | **Y** | **Y** | **U** | **Y** | **Y** | 7/8 | (87.5%)  Good | Included |

*****Score gained and the percentage of maximum score based on JBI appraisal for analytical cross-sectional studies (8 criteria): Moola S, Munn Z, Tufanaru C, Aromataris E, Sears K, Sfetcu R, Currie M, Qureshi R, Mattis P, Lisy K, Mu P-F. Chapter 7: Systematic reviews of etiology and risk. In: Aromataris E, Munn Z (Editors). JBI Manual for Evidence Synthesis. JBI, 2020. Available from <https://synthesismanual.jbi.global>

***Answers:*** **Y=YES, N=NO, U =UNCLEAR, NA=NOT APPLICABLE, NR= NOT REPORTED**

***Total score:*** Number of YES; (“YES”=1, “NO”=0, “Other; (U/ NA/NR)” = 0) and calculated the percentage of the total score.

***The quality rating:*** 67-100 **(Good)**, 34-66 **(Fair)**, and 0-33 **(Poor)**.

**Questions (8 criteria):**

**1**. Were the criteria for inclusion in the sample clearly defined?

**2**. Were the study subjects and the setting described in detail?

**3**. Was the exposure measured in a valid and reliable way?

**4**. Were objective, standard criteria used for measurement of the condition?

**5**. Were confounding factors identified?

**6**. Were strategies to deal with confounding factors stated?

**7**. Were the outcomes measured in a valid and reliable way?

**8**. Was appropriate statistical analysis used?
