# Supplementary material for: Respiratory function in healthy long-term meditators: a systematic review
Source: Syst Rev. 2024 Jan 2;13:1. doi: 10.1186/s13643-023-02412-0 (PMC10759765; doi:10.1186/s13643-023-02412-0)
Supplement: Supplementary file 6 — Additional file 6: Supplementary Table S4. Quality Assessment Tool for Studies of Single Subject Study Design. [file 13643_2023_2412_MOESM6_ESM.docx]

**Additional file 6: Supplementary Table S4:**

**Quality Assessment Tool for Studies of Single Subject Study Design**

| **The Single-Case Reporting Guideline In BEhavioural Interventions (SCRIBE) 2016 Checklist** | | | | | |  |
| --- | --- | --- | --- | --- | --- | --- |
| Item number | Topic | Item description | (Telles and Desiraju, 1993) | | (Wallance and Benson, 1972) | (Telles, Nagarathna and Nagendra, 1995) |
| **TITLE & ABSTRACT** | |  |  | |  |  |
| 1 | Title | Identify the research as a single-case experimental design in the title | NO | | NO | NO |
| 2 | abstract | Summarize the research question, population, design, methods including interventions (independent variable/s) and target behavior/s and any other outcome/s (dependent variable/s), results, and conclusions | **YES** | | NO | **YES** |
| **INTRODUTION** | |  |  | |  |  |
| 3 | Scientific background | Describe the scientific background to identify issues under analysis, current scientific knowledge, and gaps in that knowledge base | **YES** | | **YES** | **YES** |
| 4 | Aims | State the purpose/aims of the study, research question/s, and, if applicable, hypotheses | **YES** | | **YES** | **YES** |
| **METHOD**  Design | |  |  | |  |  |
| 5 | Design | Identify the design (e.g., withdrawal/reversal, multiple-baseline, alternating-treatments, changing-criterion, some combination thereof, or adaptive design) and describe the phases and phase sequence (whether determined a priori or data-driven) and, if applicable, criteria for phase change | NO | | NO | NO |
| 6 | Procedural changes | Describe any procedural changes that occurred during the course of the investigation after the start of the study | NO | | NO | NO |
| 7 | Replication | Describe any planned replication | **YES** | | NO | NO |
| 8 | Randomization | State whether randomization was used, and if so, describe the randomization method and the elements of the study that were randomized | NO | | NO | NO |
| 9 | Blinding | State whether blinding/masking was used, and if so, describe who was blinded/masked | NO | | NO | NO |
| Participants/Units | |  |  | |  |  |
| 10 | Selection criteria | State the inclusion and exclusion criteria, if applicable, and the method of recruitment | **YES** | | NO | **YES** |
| 11 | Participant characteristics | For each participant, describe the demographic characteristics and clinical (or other) features relevant to the research question, such that anonymity is ensured | **YES** | | **YES** | **YES** |
| Context | |  |  | |  |  |
| 12 | Setting | Describe characteristics of the setting and location where the study was conducted | NO | | **YES** | **YES** |
| Approvals | |  |  | |  |  |
| 13 | Ethics | State whether ethics approval was obtained and indicate if and how informed consent and/or assent were obtained | **YES** | | NO | **YES** |
| Mesaures & materials | |  |  | |  |  |
| 14 | Measures | Operationally define all target behaviors and outcome measures, describe reliability and validity, state how they were selected, and how and when they were measured | **YES** | | **YES** | **YES** |
| 15 | Equipment | Clearly describe any equipment and/or materials (e.g., technological aids, biofeedback, computer programs, intervention manuals or other material resources) used to measure target behavior/s and other outcome/s or deliver the interventions | **YES** | | **YES** | **YES** |
| Interventions | |  |  | |  |  |
| 16 | Intervention | Describe the intervention and control condition in each phase, including how and when they were actually administered, with as much detail as possible to facilitate attempts at replication | **YES** | | **YES** | **YES** |
| 17 | Procedural fidelity | Describe how procedural fidelity was evaluated in each phase | NO | | NO | NO |
| Analysis | |  |  | |  |  |
| 18 | Analyses | Describe and justify all methods used to analyze data | **YES** | | NO | **YES** |
| **RESULTS** | |  |  | |  |  |
| 19 | Sequence completed | For each participant, report the sequence actually completed, including the number of trials for each session for each case. For participant/s who did not complete, state when they stopped and the reasons | **YES** | | **YES** | **YES** |
| 20 | Outcomes and estimation | For each participant, report results, including raw data, for each target behavior and other outcome/s | **YES** | | **YES** | **YES** |
| 21 | Adverse events | State whether or not any adverse events occurred for any participant and the phase in which they occurred | NO | | NO | NO |
| **DISCUSSION** | |  |  | |  |  |
| 22 | Interpretation | Summarize findings and interpret the results in the context of current evidence | **YES** | | **YES** | **YES** |
| 23 | Limitations | Discuss limitations, addressing sources of potential bias and imprecision | NO | | NO | NO |
| 24 | Applicability | Discuss applicability and implications of the study findings | **YES** | | **YES** | **YES** |
| **DOCUMENTATION** | |  |  | |  |  |
| 25 | Protocol | If available, state where a study protocol can be accessed | NO | | NO | NO |
| 26 | Funding | Identify source/s of funding and other support; describe the role of funders | NO | | NO | **YES** |
|  | **Total score** | Number of items with affirmative answer “YES” | **15/26** | | **11/26** | **15/26** |
|  | **Quality rating** |  | | **Fair**  **(57.7%)** | **Fair**  **(42.3%)** | **Fair**  **(57.7%)** |

***Total score:*** Number of **YES**; (“**YES**”=1, “NO”=0) and calculated the percentage of the total score.

***The quality rating:*** 67-100 **(Good)**, 34-66 **(Fair)**, and 0-33 **(Poor)**.
